# Mitochondrial-Targeted Protective Potential of Elamipretide for the In Vitro Production of Porcine Embryos

**DOI:** 10.3390/ani15172497

**Published:** 2025-08-25

**Authors:** Suong T. Nguyen, Takeshige Otoi, Zhao Namula, Oky Setyo Widodo, Theerawat Tharasanit, Kaywalee Chatdarong, Yuichiro Nakayama, Megumi Nagahara, Aya Nakai, Maki Hirata, Fuminori Tanihara

**Affiliations:** 1 Bio-Innovation Research Center, Tokushima University, Tokushima 7793233, Japan; suongnt268@gmail.com (S.T.N.); otoi@tokushima-u.ac.jp (T.O.); c702101052@tokushima-u.ac.jp (Y.N.); nagahara@tokushima-u.ac.jp (M.N.); nakai.aya.1@tokushima-u.ac.jp (A.N.); mhirata@tokushima-u.ac.jp (M.H.); 2 Faculty of Veterinary Medicine, Universitas Airlangga, Surabaya 60115, Indonesia; oky.widodo@fkh.unair.ac.id; 3 Faculty of Veterinary Science, Chulalongkorn University, Bangkok 10330, Thailand; theerawat.t@chula.ac.th (T.T.); kaywalee.c@chula.ac.th (K.C.); 4 Department of Veterinary Medicine, College of Coastal Agricultural Sciences, Guangdong Ocean University, Zhanjiang 524088, China; zhaonamula@gdou.edu.cn

**Keywords:** elamipretide, embryo, glutathione, mitochondria, porcine, reactive oxygen species

## Abstract

Mitochondrial-targeted antioxidant supplementation is promising for in vitro culture of mammalian embryos. Elamipretide (SS-31) is a synthetic tetrapeptide that binds to the inner mitochondrial membrane, contributing to the prevention of oxidative stress. In this study, the effects of SS-31 supplementation in maturation medium on the developmental competence of porcine oocytes and its protective function were evaluated. Porcine oocytes were matured in maturation medium with SS-31 at 0.001, 0.01, 0.1, 0.5, 1, 1.5, 2.5, and 5 µM, with 0 µM and DMSO-treated groups established as controls. In vitro fertilization and embryo culture were performed to analyze developmental potential. Oocytes cultured in medium with 1 µM SS-31 exhibited higher maturation and blastocyst formation rates than the control (0 µM) (78.3 ± 3.8% vs. 55.2 ± 4.1% and 7.6 ± 1.6% vs. 2.8 ± 1.8%, respectively). Oocytes treated with 1 µM SS-31 showed significantly lower reactive oxygen species, higher glutathione content, and improved mitochondrial membrane potential than those without treatment. DNA fragmentation in oocytes after in vitro maturation was significantly lower in the 1 µM SS-31 supplemented group than in the control. This study demonstrates that SS-31 exerts beneficial effects on the in vitro production of porcine embryos via the enhancement of the mitochondrial antioxidant capacity.

## Simple Summary

Excessive production of reactive oxygen species during in vitro embryo production can impair mitochondrial defense mechanisms, thereby reducing embryonic developmental competence. We investigated the effects of elamipretide, a mitochondrial-targeted antioxidant, on porcine oocyte maturation and subsequent embryo development in vitro. The results demonstrated that SS-31 enhanced the antioxidative capacity of oocytes by reducing intracellular reactive oxygen species levels, increasing glutathione content, improving mitochondrial membrane potential, and reducing DNA fragmentation, resulting in higher rates of oocyte maturation and blastocyst formation.

## 1. Introduction

Mitochondria play a key role in oocyte development and maturation by maintaining intracellular calcium (Ca^2+^) homeostasis and acting as messengers for many physiological processes. They are the main source of adenosine-5-triphosphate (ATP) through the oxidative phosphorylation (OXPHOS) system [1,2,3]. Mitochondrial development is a dynamic process reflecting the energy demand at each stage of oocyte growth. This process becomes particularly important during the transition from the germinal vesicle stage to maturation due to the sharp increase in energy consumption for events such as germinal vesicle breakdown, mitotic spindle migration, and polar body extrusion [4], requiring an increase in mitochondrial size and density, distributed in clusters throughout the cytoplasm [5]. The developmental competence of oocytes/embryos is highly dependent on mitochondria, and mitochondrial loss or dysfunction is associated with reduced oocyte quality [6]. Accordingly, improving oocyte quality through treatments aimed at mitochondrial enhancement has attracted substantial attention in the development of in vitro production systems [7,8]. 

Reactive oxygen species (ROS) are a major risk factor for mitochondrial dysfunction, inhibiting oocyte and embryonic development. Under normal physiological conditions, ROS are byproducts of oxidative metabolism, generated primarily through the activity of mitochondrial respiratory complexes I, II, and III, which are most abundant in the mitochondria of mature oocytes [9,10,11]. To maintain redox homeostasis, oocytes activate ROS-neutralizing mechanisms by upregulating endogenous non-enzymatic and enzymatic antioxidants, such as glutathione (GSH), glutathione peroxidase, superoxide dismutase, and catalase [12,13]. However, suboptimal culture medium and in vitro oocyte handling often lead to excessive ROS production [13,14,15]. When the ROS burden exceeds the cellular antioxidant defense capacity, it can disrupt mitochondrial physiology, influence Ca^2+^ signaling, and induce the opening of mitochondrial permeability transition pore channels, resulting in proton leak and membrane depolarization [16,17,18]. 

To counteract mitochondrial oxidative stress, mitochondria-targeted antioxidants have been developed to selectively accumulate at the primary site of ROS generation. This targeted delivery improves antioxidant bioavailability, enabling therapeutic effects at lower doses compared to non-specific intracellular accumulation, which typically requires much higher concentrations and is associated with increased risk of side effects [3,19,20]. In contrast, given that mitochondrial oxidative events precede those in the cytoplasm, non-targeted antioxidants can traverse physiological barriers but typically fail to penetrate mitochondria membranes, limiting their capacity to effectively neutralize ROS at the source [21]. Among mitochondria-targeted agents, Szeto–Schiller (SS) peptides are a class of small, water-soluble, positively charged peptides capable of diffusing across cellular and mitochondrial membranes, accumulating 1000–5000-fold within mitochondria. Of the identified variants, such as SS-02, SS-20, and SS-31 (Elamipretide, D-Arg-Dmt-Lys-Phe-NH2), SS-31 has demonstrated the most promising therapeutic potential [22,23].

SS-31 selectively accumulates on the inner mitochondrial membrane, where it reduces ROS production. Designed to cross the blood–brain barrier, SS-31 supports mitochondrial membrane integrity and optimizes electron transport and ATP synthesis [24,25]. There is abundant evidence supporting the protective effects of SS-31 against various inflammatory diseases [26,27], age-related damage [28,29,30], and cytotoxicity [31,32]. Moreover, SS-31 is considered a safe compound, as it selectively targets supraphysiological levels of ROS without interfering with physiological ROS levels [33]. This property makes it a promising candidate for treating diseases associated with mitochondrial dysfunction. Although SS-31 has demonstrated mitochondrial protective effects in several cell types, its effects on in vitro porcine oocyte culture systems have not been investigated.

We hypothesized that SS-31 would improve the outcomes of in vitro porcine embryo production. In this study, we first evaluated the effects of SS-31 supplementation in the porcine in vitro maturation (IVM) medium on oocyte maturation and subsequent blastocyst formation. Next, the antioxidant capacity of SS-31 was assessed using indicators of mitochondrial function, including ROS, GSH levels, and mitochondrial membrane potential (ΔΨm). In addition, DNA fragmentation was evaluated in cultured oocytes.

## 2. Materials and Methods

### 2.1. Ethical Approval

No live animals were used in this study.

### 2.2. Collection and In Vitro Culture of Porcine Oocytes 

The procedures for oocyte collection and IVM followed those of a previous study [34], with minor modifications. Briefly, ovaries were obtained from prepubertal crossbred gilts (Landrace × Large White × Duroc breeds) at a local slaughterhouse and transferred to the laboratory within 2 h in 0.9% (*w*/*v*) physiological saline at (30–35 °C). After washing the ovaries three times with physiological saline solution containing 100 IU/mL of penicillin G potassium (Meiji Seika Pharma, Tokyo, Japan) and 0.1 mg/mL of streptomycin sulfate (Meiji Seika Pharma), cumulus–oocyte complexes (COCs) were collected from follicles (3–6 mm in diameter) using the slicing method with a surgical blade within no more than 30 min, within approximately 4 h after slaughter. Only COCs with a uniform dark-pigmented ooplasm and intact cumulus cell mass were selected for this experiment. The maturation culture medium was composed of tissue culture medium 199 with Earle’s salts (TCM 199; Thermo Fisher Scientific, Waltham, MA, USA) supplemented with 10% (*v*/*v*) porcine follicular fluid, 50 µg/mL of gentamicin (G3632, Sigma-Aldrich, St. Louis, MO, USA), 50 µM sodium pyruvate (P5280, Sigma-Aldrich), 2 mg/mL of D-sorbitol (S7547, Sigma-Aldrich), 10 IU/mL of equine chorionic gonadotropin (Kyoritsu Seiyaku, Tokyo, Japan), and 10 IU/mL of human chorionic gonadotropin (Kyoritsu Seiyaku). Approximately 50 COCs were transferred to 500 µL of culture medium covered with mineral oil (M8410, Sigma-Aldrich) in a four-well dish (Thermo Fisher Scientific) and incubated for 22 h at (39 °C) with 5% CO_2_. Thereafter, COCs were cultured for 22 h in the mature medium without equine and human chorionic gonadotropins. Approximately 30% of COCs in each group were randomly collected after 44 h of maturation culture and subjected to further assessment.

### 2.3. Analysis of Oocyte Nuclear Maturation

After maturation culture, COCs were denuded from cumulus cells using 150 IU/mL of hyaluronidase (H3506, Sigma-Aldrich), followed by mechanical pipetting. The oocytes were fixed in 3.7% (*w*/*v*) paraformaldehyde phosphate buffer (FUJIFILM Wako Pure Chemical Corporation, Osaka, Japan) at (4 °C) overnight and subsequently permeabilized with 0.1% Triton™ X-100 (T8787, Sigma-Aldrich) for 1 h at (25 °C). They were then treated with 1.9 mM bisbenzimide (Hoechst 33342; Sigma-Aldrich), mounted on a glass slide, and sealed with clear nail polish. Labeled oocytes were examined using a microscope (Olympus, Optical Co., Tokyo, Japan) equipped with an epi-fluorescence excitation filter at 355 nm. 

### 2.4. Assessment of ROS and GSH Levels in Oocyte Cytoplasm After Maturation Culture

The intra-oocyte levels of ROS and GSH were assessed following the method described by Yousefian et al. [35], with minor modifications. Denuded oocytes were incubated in IVM medium containing 10 µM 2′,7′-dichlorodihydrofluorescein diacetate (H2DCF-DA, D399; Thermo Fisher Scientific) and 10 µM 4-chloromethyl-6,8-difluoro-7-hydroxycoumarin (CellTracker™ Fluorescent Probes CMF2HC, C12881; Thermo Fisher Scientific) at (38.5 °C), with 5% CO_2_ in the dark for 15 and 30 min to assess ROS and GSH levels, respectively. After incubation, oocytes were washed and transferred to 10 µL drops of IVM medium. Images of fluorescently labeled oocytes were captured using the BZ-X710 fluorescence microscope (KEYENCE, Osaka, Japan) and saved as TIFF files. Two standard filter sets were used for the detection of ROS (excitation wavelength of 492–495 nm and emission wavelength of 517–527 nm) and GSH (excitation wavelength of 371 nm and emission wavelength of 464 nm). Fluorescence intensities were analyzed using ImageJ software (version 1.54g; National Institutes of Health, Bethesda, MD, USA). The area of fluorescence intensity measured on the image was selected as the region of interest, and the mean intensity (mean) in the area was recorded for each assessment. 

### 2.5. Assessment of Mitochondrial Membrane Potential in Oocytes After Maturation Culture

Oocyte mitochondria membrane potential (ΔΨm) was assessed according to the method of Lee et al. [36], with a slight modification, using 5′,6,6′-tetrachloro-1,1′,3,3′-tetraethylbenzimidazolylcarbocyanine iodide (MitoProbe™ JC-1 Assay; Thermo Fisher Scientific). Briefly, denuded oocytes were washed using IVM medium and fixed in 3.7% (*w*/*v*) paraformaldehyde phosphate buffer for 15 min at (25 °C). The fixed oocytes were washed in DPBS (FUJIFILM Wako Pure Chemical Corporation) containing 0.3% (*w*/*v*) polyvinyl alcohol (P8136, Sigma-Aldrich) (DPBS/PVA) and then co-incubated with IVM medium containing 2 µM JC-1 for 30 min. Next, oocytes were re-washed in DPBS/PVA and mounted on glass slides. Images of fluorescently labeled oocytes were captured using the BZ-X710 fluorescence microscope; the green monomeric form had absorption/emission wavelengths of 510/529 nm, and J-aggregate red forms had absorption/emission wavelengths of 585/590 nm, respectively. Images were saved as TIFF files, and green and red fluorescence intensity of each oocyte was measured using ImageJ software. The relative red (high ΔΨm/JC-1 aggregates) to green (low ΔΨm/JC-1 monomers) fluorescence ratio was measured to assess mitochondrial membrane potential.

### 2.6. Detection of DNA-Fragmented Oocytes After Maturation Culture

After IVM, cultured oocytes were analyzed for DNA fragmentation using terminal deoxynucleotidyl transferase nick-end labeling (TUNEL) staining, following the protocol described by Lin et al. [37]. Cumulus cells were removed by incubating the samples with 150 IU/mL of hyaluronidase at (37 °C) combined with gentle pipetting. The denuded oocytes were fixed overnight at (4 °C) in 3.7% (*w*/*v*) paraformaldehyde, permeabilized for 1 h at (25 °C) with 0.1% Triton X-100, and incubated overnight at (4 °C) in blocking solution containing phosphate-buffered saline with 10 mg/mL bovine serum albumin (A9647, Sigma-Aldrich). For TUNEL labeling, oocytes were incubated with fluorescein-conjugated 2-deoxyuridine-5′-triphosphate and TUNEL reagent (In Situ Death Detection Kit Fluorescein, Roche, Germany) for 1 h at (38 °C). After labeling, nuclei were treated with an anti-fade solution (Fluoro-KEEPER Antifade Reagent, Non-Hardening Type with DAPI, Nacalai tesque, Kyoto, Japan). Oocytes were mounted on glass slides, sealed with clear nail polish, and examined using an Eclipse 80i epifluorescence microscope (Nikon, Tokyo, Japan). The DNA fragmentation rate was expressed as the proportion of oocytes with DNA-fragmented nuclei relative to the total number examined. DNA-fragmented nuclei were not observed in degenerated oocytes.

### 2.7. In Vitro Fertilization and In Vitro Culture

After IVM, oocytes were washed with porcine fertilization medium (PFM; Functional Peptide Institute, Yamagata, Japan). Approximately 60 oocytes per well were transferred to a four-well dish filled with 600 µL of PFM per well and covered with mineral oil, containing frozen–thawed spermatozoa at a final concentration of 1 × 10^6^ sperm/mL at (39 °C), 5% CO_2_, and 5% O_2_. Sperm was prepared by thawing at (38 °C) for 30 s, followed by washing through centrifugation with PFM at (25 °C), 500× *g* for 5 min, and then diluted in PFM to the desired concentration. After 5 h of co-incubation, cumulus cells and loosely attached spermatozoa were removed from the presumed zygotes via gentle mechanical pipetting and cultured in porcine zygote medium (PZM-5; Research Institute for the Functional Peptides Co. Yamagata, Japan) for 72 h at (39 °C), with 5% CO_2_, 5% O_2_, and 90% N_2_. Then, these embryos were cultured in porcine blastocyst medium (Research Institute for the Functional Peptides Co.) for 4 days to assess the rate of development. The number of cells per blastocyst was assessed as described previously [37].

### 2.8. Experimental Design

#### 2.8.1. Experiment 1. Effects of SS-31 Supplementation on the IVM of Oocytes and Subsequent Embryonic Development

To evaluate the effects of SS-31 on oocyte maturation and embryonic development, SS-31 (MTP-131, Selleck Chemicals, Houston, TX, USA) was added to IVM medium at various concentrations (0.001, 0.01, 0.1, 0.5, 1, 1.5, 2.5, and 5 µM). As a control, COCs were cultured in maturation medium either without SS-31 and its dilution vehicle (dimethyl sulphoxide [DMSO]) or without SS-31 only. After IVM, approximately 30% of the oocytes from each group were randomly collected and used to assess the maturation rate. The remaining oocytes were subjected to in vitro fertilization and embryo culture. The cleavage rate was assessed on day 3 after fertilization, and blastocyst formation was evaluated on day 7 after fertilization.

#### 2.8.2. Experiment 2. Effects of SS-31 Supplementation on Antioxidant Capacity and Mitochondrial Membrane Potential After IVM

To clarify the antioxidant activity of SS-31, the optimal concentration of SS-31 (1 µM), determined based on the results of experiment 1, was used to verify its effects on intracellular ROS and GSH levels. In addition, the mitochondrial membrane potential was assessed in oocytes after IVM. As a control, COCs were cultured in maturation medium without SS-31.

#### 2.8.3. Experiment 3. Effect of SS-31 Supplementation on Oocyte DNA Fragmentation After IVM 

To evaluate the protective effect of SS-31 against DNA fragmentation, an indicator of apoptosis, oocytes were cultured in IVM medium supplemented with 1 µM SS-31 or without supplementation (control). Following the maturation period, 20 oocytes from each group were randomly selected for DNA fragmentation analysis using the TUNEL assay. 

### 2.9. Statistical Analysis

Data expressed as percentages for maturation, cleavage, and blastocyst rates were converted to arcsine values before statistical analysis. Relative values of ROS, GSH, and JC-1 in the experimental group are expressed as relative changes compared with values in the control group normalized to 1-fold. Statistical differences were assessed using one-way analysis of variance, followed by Fisher’s protected least significant difference test using STATVIEW software (Abacus Concepts, Inc., Berkeley, CA, USA). Data are expressed as the mean ± standard error of the mean (SEM). Differences were considered significant at a probability value of *p* ≤ 0.05.

## 3. Results

### 3.1. Experiment 1. Effects of SS-31 Supplementation on the IVM of Oocytes and Subsequent Embryonic Development 

The IVM rate, as indicated by the percentage of oocytes reaching the MII stage, was significantly higher in the group supplemented with 1 µM SS-31 than in the other groups, with the exception of the 0.1 µM and 0.5 µM SS-31 groups (*p* < 0.05) (Table 1). The highest maturation rate was observed at 1 µM SS-31, whereas further increases to 1.5, 2.5, and 5 µM resulted in a significant decline. The cleavage rates in the 2.5 and 5 µM groups were lower than those in the control and DMSO groups (*p* < 0.05). The blastocyst formation rate was significantly higher in the 1 µM SS-31 group than in the control and DMSO groups (*p* < 0.05). The number of cells per blastocyst did not differ significantly among groups. Based on these results, 1 µM SS-31 was selected for subsequent analyses. 

### 3.2. Experiment 2. Effects of SS-31 Supplementation on Antioxidant Capacity and Mitochondrial Membrane Potential After IVM

**Figure 1:**
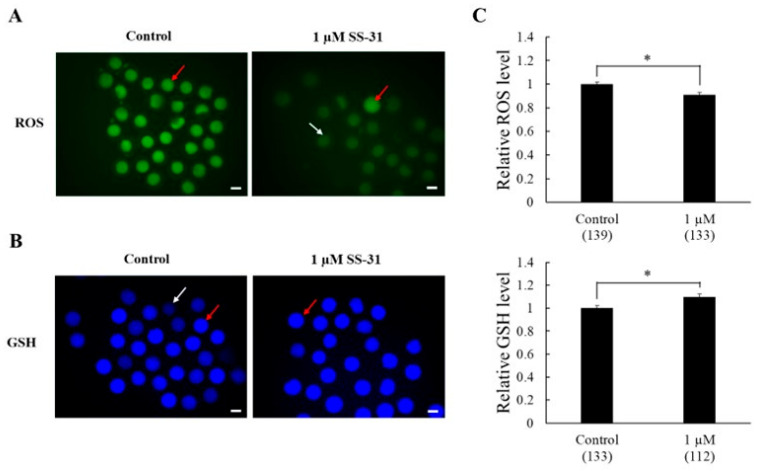
Effects of SS-31 on antioxidant capacity in oocytes after in vitro maturation. (**A**) Fluorescence intensity of DCFH-DA green-stained oocytes indicating the reactive oxygen species (ROS) content. (**B**) Fluorescence intensity of CMF2HC blue-stained oocytes representing glutathione (GSH) production. The white arrow indicates a low-ROS and -GSH oocyte, and the red arrow indicates a high-ROS and -GSH oocyte. (**C**) Total ROS and GSH levels in oocytes were analyzed using ImageJ. Four replicates were performed. Means ± SEM are presented for all data. Asterisks indicate statistical significance (* *p* < 0.01). Numbers within parentheses indicate the total number of oocytes examined. Scale bar indicates 100 µm.

Compared to the control group, oocytes treated with 1 µM SS-31 exhibited decreased green fluorescence intensity, indicating significantly lower ROS levels (0.91 ± 0.023-fold vs. 1.00 ± 0.016; *p <* 0.01, Figure 1A,C). GSH content was significantly higher in the SS-31 treated oocytes than in the control group, as evidenced by increased blue fluorescence (1.10 ± 0.025-fold vs. 1.00 ± 0.02; *p <* 0.01, Figure 1B,C).

**Figure 2:**
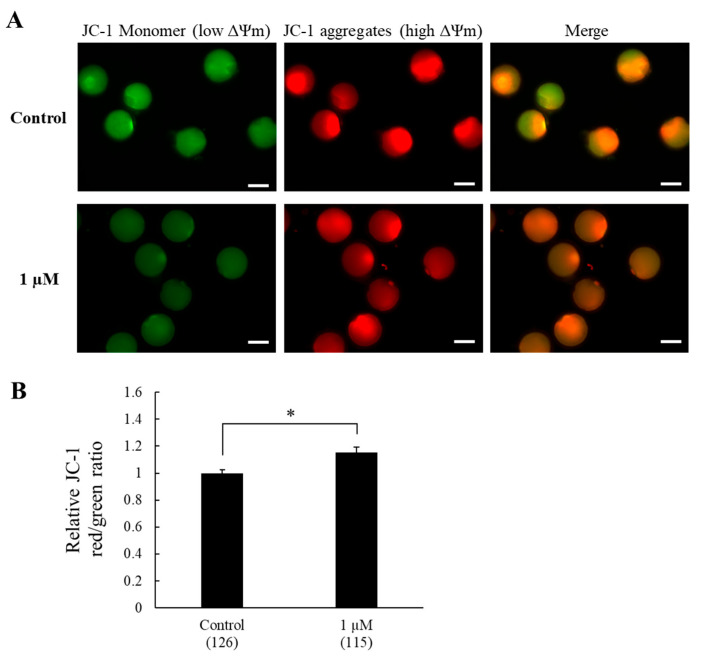
Effects of SS-31 on mitochondrial membrane potential (ΔΨm) in JC-1-labeled oocytes. (**A**) Representative images of oocyte depicting JC-1. (**B**) Relative ΔΨm expressed as the red-to-green intensity ratio. Green: mitochondria with low ∆Ψm; red: mitochondria with high ∆Ψm; yellow: merging of green and red images. Means ± SEM are presented of all data. The experiment was replicated four times. Asterisks indicate statistical significance (* *p* < 0.01). Numbers within parentheses indicate the total number of oocytes examined. Scale bar indicates 100 µm.

Mitochondrial membrane potential was assessed based on the fluorescence shift of JC-1 dye from green (monomers) to red (J-aggregates). A greater shift from green to red fluorescence indicates increased accumulation of J-aggregates, reflecting higher mitochondrial membrane potential and vice versa. The results showed that oocytes in the control group exhibited higher green fluorescence and lower red shift, indicating reduced J-aggregate formation (i.e., a predominance of JC-1 monomers) and lower mitochondrial membrane potential. In contrast, oocytes treated with 1 µM SS-31 displayed a stronger red shift, reflecting increased J-aggregate accumulation and higher mitochondrial membrane potential (Figure 2A). Consistently, the relative red-to-green fluorescence ratio in oocytes treated with 1 µM SS-31 was significantly higher than that in the control (1.15 ± 0.04-fold vs. 1.00 ± 0.03, *p <* 0.01, Figure 2B), confirming enhanced mitochondrial function.

### 3.3. Experiment 3. Effect of SS-31 Supplementation on DNA-Fragmented Nuclei in Oocytes After IVM 

**Figure 3:**
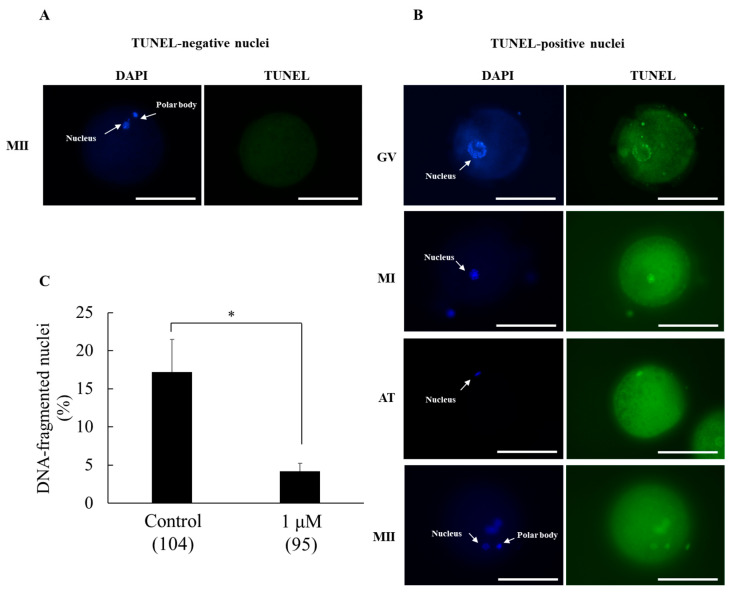
Effect of SS-31 supplementation on DNA fragmentation in oocyte nuclei after in vitro maturation. (**A**) Representative image of an MII-stage oocyte without signs of DNA fragmentation. (**B**) Representative images of DNA-fragmented oocytes at various developmental stages: germinal vesicle (GV), metaphase I (MI), anaphase I/telophase I (AT), and metaphase II (MII). DAPI staining visualizes nuclei (blue), whereas TUNEL staining detects DNA fragmentation (green). (**C**) Percentage of oocytes with DNA-fragmented nuclei, presented as mean ± SEM. The experiment was replicated five times. Numbers within parentheses indicate the total number of oocytes examined. Asterisks indicate statistical significance (* *p* < 0.05). Scale bar indicates 100 µm.

The potential protective effect of SS-31 supplementation to IVM medium against DNA fragmentation, an indicator of apoptosis, was evaluated. Non-DNA-fragmented oocytes exhibited DAPI-positive (blue) nuclei with no TUNEL staining (absence of green fluorescence) (Figure 3A). In contrast, DNA-fragmented oocytes were positive for both DAPI (blue) and TUNEL (green) staining (Figure 3B). Compared with the control, treatment with 1 µM SS-31 significantly reduced the DNA fragmentation rate (4.2 ± 1.0% vs. 17.2 ± 4.3%, *p <* 0.05) (Figure 3C). 

## 4. Discussion

In vitro production systems have been established for porcine embryos; however, morphologically normal oocytes reaching a developmental stage suitable for fertilization and subsequent embryonic development are limited. Previous studies have highlighted the detrimental effects of oxidative stress on oocytes during in vitro culture [14,15]. Mitochondrial function is vulnerable to such stress, often resulting in increased intracellular ROS; impaired cellular respiration and ATP biosynthesis; and ultimately, cell death. Therefore, enhancing mitochondrial function has emerged as a promising therapeutic approach in mitochondrial disorders. 

SS-31 is a mitochondrial-targeted antioxidant compound. To our knowledge, this is the first study to investigate the effects of SS-31 supplementation on a porcine in vitro production system. Our results demonstrated that SS-31 enhances oocyte maturation efficiency and subsequent blastocyst formation. A study of female germ cells reported that SS-31 treatment of mouse ovarian tissue during vitrification and cryo-resuscitation improves follicle and oocyte quality, reduces oxidative stress, preserves ATP levels, and increases mitochondrial DNA copy number [38]. These results are consistent with those of the present study, despite differences in the SS-31 treatment methods and animal models. In male germ cells, the protective effects of SS-31 against cryopreservation and freeze–thaw injury have also been demonstrated in both humans and bulls [39,40]. A major advantage of SS-31 over other mitochondria-targeted antioxidants is its membrane potential-independent uptake [23]. In contrast, antioxidants such as mitoquinone [2], SkQ1 [41], and MitoTEMPO [35,42], although beneficial for in vitro embryo development, require a delivery vector (triphenylphosphonium cation) to selectively accumulate in mitochondria in a membrane potential-dependent manner [43]. This dependence can hinder their therapeutic consistency under variable physiological or pathological conditions. N-acetyl-L-cysteine [44] and quercetin [45] require liposomal encapsulation to facilitate cellular uptake via lipid fusion, phagocytosis, or receptor-mediated endocytosis, thereby prolonging their half-life and enhancing bioavailability [46]. A next-generation liposomal nanocarrier system, known as the Mito-porter, has been developed to enable direct mitochondrial delivery via membrane fusion [47], but its efficacy in improving in vitro embryo production remains to be established.

Although SS-31 treatment during the IVM period improved the blastocyst formation rate by enhancing oocyte quality, no significant difference was observed in the average cell number of the resulting blastocysts. This may be attributed to the stage-dependent effects of antioxidant supplementation, as previously reported [48]. In the present study, the beneficial effects of SS-31 appeared to be primarily exerted during the early stages of embryonic development, from oocyte maturation to fertilization and early cleavage. Conversely, supplementation with SS-31 during in vitro culture, especially at later stages of embryonic development, has the potential to improve or otherwise modulate blastocyst quality. Therefore, further studies are warranted to investigate the effects of SS-31 supplementation during the in vitro culture period on blastocyst quality. Additionally, in this study, the blastocyst rate was notably low. As the primary aim was to evaluate the antioxidant effect of SS-31 during IVM, commonly used antioxidants were intentionally excluded. Given the well-documented sensitivity of porcine oocytes to oxidative stress, the absence of antioxidant supplementation likely contributed to the reduced embryonic development observed, which is consistent with our previous findings [49].

The effect of SS-31 was dose-dependent. Supplementation with 1 µM SS-31 enhanced oocyte quality and developmental competence, whereas the highest concentration (5 µM) significantly impaired oocyte maturation, cleavage, and the blastocyst formation rate. This observation is consistent with a previously proposed mechanism [50] suggesting that high doses of SS-31 can alter lipid packing, diffusion, and mitochondrial membrane surface charge, despite an intact mitochondrial bilayer structure, ultimately affecting oocyte maturation.

We further evaluated the contribution of antioxidative activity to the effects of SS-31 on oocyte mitochondria through comparisons of oxidative stress indicators between the 1 µM treatment group, identified as optimal in the first experiment, and the control group without SS-31. The results demonstrated that SS-31 treatment promoted ROS scavenging and GSH synthesis. This may be explained by the mechanism through which SS-31 acts on its target organelles. Mitochondria are double-membrane organelles, with the inner membrane forming invaginations known as cristae, where cardiolipin (CL), a unique phospholipid, is concentrated. CL depletion due to oxidative stress can disrupt mitochondrial function, causing cell death [51]. SS-31 is a cell-permeable tetrapeptide that combines CL in the inner mitochondrial membrane [50], contributing to a reduction in CL peroxidation. It also mitigates ROS production by inhibiting cytochrome c peroxidase release, promoting the assembly and stabilization of mitochondrial respiratory complexes I and III on the mitochondrial membrane, which limits electron leakage, a major source of mitochondrial ROS [23,52,53,54]. In addition, Ca^2+^ influx and storage in mitochondria are closely associated with CL dynamics [55,56]. Under calcium stress, SS-31 reduces Ca^2+^ accumulation on the surface of the mitochondrial bilayer, helping to maintain oxidative phosphorylation and membrane integrity [50]. The present study also demonstrated that the mitochondrial protection was significantly enhanced by SS-31 supplementation, as evidenced by increased levels of intra-oocyte GSH, a well-known antioxidant involved in ROS detoxification [57]. Similar conclusions were drawn from observations of aged mouse mitochondria, when SS-31 treatment resulted in an increase in GSH [53].

In the present study, the mitochondrial membrane potential of oocytes after SS-31 treatment was assessed using JC-1 [58]. This is a lipophilic cationic dye that initially exists in monomeric form and exhibits green fluorescence and converts to red fluorescent J-aggregates after accumulation in negatively charged mitochondria with an emission level dependent on the mitochondrial membrane potential. As a loss of membrane potential causes a decrease in mitochondrial negative potential, insufficient J-aggregate formation results in a decrease in red or continued green fluorescence. In this study, a higher mitochondrial membrane potential with a significantly higher red/green ratio, along with reduced ROS generation, was found under SS-31 treatment compared with that in the control group. Similar results were found in aged cardiomyocytes, in which SS-31 promoted reduced proton leak and reduced opening of the mitochondrial permeability transition, thus enhancing mitochondrial membrane potential [57]. Furthermore, mitochondrial dysfunction and oxidative stress contribute to increased oocyte apoptosis [59]. Accordingly, we investigated the antioxidant potential of SS-31 in mitigating DNA fragmentation in oocytes. Our results demonstrated that supplementation with optimal concentrations of SS-31 significantly reduced the DNA fragmentation rate compared with that in the untreated controls, consistent with previous findings [38]. This reduction will be associated with improved oocyte maturation rates and enhanced subsequent blastocyst formation.

Previous cellular studies have indicated that the efficacy of SS-31 is specific to damaged mitochondria [53,60], a parameter that was not directly assessed in the present study. Porcine oocytes are highly sensitive to oxidative stress; therefore, conventional culture media are often supplemented with antioxidants such as cysteine and β-mercaptoethanol [34,37]. In the present study, these antioxidants were intentionally omitted to better elucidate the effects of SS-31, thereby creating conditions in which mitochondrial damage was likely but not quantitatively evaluated. Under these circumstances, the observed improvement in oocyte quality suggests that SS-31 may have contributed to the restoration of mitochondrial function. Future investigations will incorporate direct assessments of mitochondrial damage to strengthen the mechanistic interpretation of SS-31’s effects.

## 5. Conclusions

This study provides the first evidence for the beneficial effects of SS-31 in the in vitro production of porcine oocytes. SS-31 enhanced oocyte maturation and blastocyst formation, and its mitochondrial-targeted effects were mediated by a reduction in intra-oocyte ROS, increase in GSH content, preservation of mitochondrial membrane potential, and inhibition of DNA fragmentation. 

## Figures and Tables

**Table 1:** Effects of SS-31 supplementation on the maturation of oocytes and subsequent embryonic development.

SS-31 Concentration (µM) *	In Vitro Maturation		In Vitro Culture			
	No. of Examined Oocytes	No. (%) of Oocytes at MII Stage ** (%)	No. of Examined Oocytes	No. (%) of Cleaved Embryos ***	No. (%) of Embryos Developed to Blastocysts ****	Total Cell Number in Blastocysts
Control	87	50 (55.2 ± 4.1) ^ab^	177	131 (73.5 ± 3.9) ^a^	5 (2.8 ± 1.8) ^a^	47.4 ± 8.5
DMSO	86	48 (55.6 ± 4.2) ^ab^	201	148 (74.2 ± 2.9) ^a^	6 (3.0 ± 0.4) ^a^	32.7 ± 3.0
0.001	87	54 (60.5 ± 7.9) ^ab^	179	147 (81.8 ± 3.2) ^a^	9 (4.9 ± 1.5) ^ab^	40.0 ± 8.3
0.01	79	51 (62.7 ± 5.4) ^ab^	195	160 (81.4 ± 3.5) ^a^	7 (3.6 ± 0.6) ^ab^	46.3 ± 9.5
0.1	80	55 (67.2 ± 5.4) ^ac^	185	145 (77.8 ± 3.5) ^ab^	8 (4.0 ± 1.9) ^ab^	42.1 ± 10.1
0.5	91	63 (69.6± 5.4) ^ac^	205	160 (78.0 ± 3.3) ^ab^	10 (4.9 ± 1.7) ^ab^	51.2 ± 7.7
1	81	63 (78.3 ± 3.8) ^c^	190	156 (81.7 ± 3.1) ^a^	14 (7.6 ± 1.6) ^b^	43.3 ± 5.8
1.5	92	56 (60.9 ± 2.9) ^ab^	202	158 (78.2 ± 3.5) ^ab^	9 (4.5 ± 1.4) ^ab^	43.2 ± 3.6
2.5	96	61 (63.4 ± 2.5) ^ab^	194	136 (70.0 ± 4.9) ^bc^	13 (6.4 ± 1.8) ^ab^	40.7 ± 3.2
5	95	49 (50.7 ± 8.5) ^b^	199	128 (64.6 ± 1.4) ^c^	5 (2.4 ± 1.3) ^a^	51.8 ± 11.4

* As a control, COCs were cultured in maturation medium either without SS-31 and its dilution vehicle (dimethyl sulphoxide [DMSO]) or without SS-31 only. In the DMSO group, COCs were cultured in maturation medium supplemented with DMSO without SS-31. ** The maturation rate was calculated by dividing the total number of oocytes reaching the metaphase II (MII) stage by the total number of evaluated oocytes. *** The cleavage rate was determined by dividing the number of cleavage embryos by the total number of embryos. **** The blastocyst formation rate was determined by dividing the number of blastocysts by the total number of embryos. ^a–c^ Values with different superscript letters in the same column were significantly different (*p* < 0.05). Five replications were performed. Values are presented as the mean ± SEM.

## Data Availability

The datasets supporting the conclusions of this article are included within the article.

## Abbreviations

The following abbreviations are used in this manuscript:

ATP	adenosine-5-triphosphate
CL	cardiolipin
COC	cumulus–oocyte complex
DMSO	dimethyl sulfoxide
GSH	glutathione
IVM	in vitro maturation
OXPHOS	oxidative phosphorylation
PFM	porcine fertilization medium
ROS	reactive oxygen species
SS	Szeto–Schiller
